# Trans and gender-diverse people’s experiences of primary care in Sweden – a qualitative study

**DOI:** 10.1186/s12875-025-03100-9

**Published:** 2025-11-15

**Authors:** Axel Repka, Ida Linander

**Affiliations:** Department of Epidemiology and Global Health, Umeå University, Umeå, 901 87 SE Sweden

**Keywords:** Transgender, Gender-diverse, Primary care, Cisnormativity, Qualitative, Gender dysphoria, Healthcare, Sweden

## Abstract

**Background:**

Trans and gender-diverse people experience a high burden of health issues and face barriers to accessing care. Primary care has a large responsibility in providing equitable access to care and thus improving the health of the population. We, therefore, explored trans and gender-diverse people’s experiences of primary care in Sweden.

**Method:**

Semi-structured interviews were conducted with 12 self-identified trans or gender-diverse persons living in Sweden, aged over 15, and with experience of primary care. Participants were recruited via trans organizations. The interviews were analysed using reflexive thematic analysis.

**Results:**

Five themes were identified: “Preparations and low expectations”, “Attempts at trans-competent care”, “When trans experiences are not present – from smooth to excluded”, “When being trans becomes a problem – from friction to overt violations”, and “Effects of healthcare encounters – from discomfort to shifted responsibility”.

**Conclusion:**

Findings show that participants’ experiences with primary care in Sweden were influenced by cisnormativity, leading to invisibility, exclusion, misgendering, and a lack of knowledge. Experiences of discomfort on the part of care providers, ridicule, disrespect, and denial of care imply the presence of transphobia. These barriers result in trans and gender-diverse people experiencing low levels of trust, having to navigate their care without support, and not having their healthcare needs met. There is an urgent need for increased trans competence and allyship with trans communities to ensure equitable access to healthcare. Training and further research should be developed in close consultation with trans organisations to address these disparities.

**Supplementary Information:**

The online version contains supplementary material available at 10.1186/s12875-025-03100-9.

## Background

Trans and gender-diverse people are people who have a different gender identity from their sex assigned at birth, and include people who identify within and outside the binary gender system. People who identify with their sex assigned at birth are described as cisgender. Cisnormativity is a system or ideology that upholds the notion that being cisgender is normal and that gender identity is fixed throughout one’s life, thereby excluding the existence of trans and gender-diverse people in society [1]. In this paper we follow Kennedy and define cisnormativity as the cultural, social, and institutional anti-trans processes, while transphobia describes individual negative actions against trans or gender-diverse people based on fear or hatred [1].

Trans and gender-diverse people experience a high burden of health issues, which can be understood as effects of living in a cisnormative society and being exposed to transphobia, stigma, and minority stress [2–4]. They also show more negative health outcomes in many areas compared to the general population; for example, the group is reported to have a much higher prevalence of suicidal thoughts and attempts [2]. However, for people who have received gender-affirming care, suicide prevalence drops to levels similar to the rest of the population [5, 6]. For many years, the queues for gender-affirming care in Sweden have been long, often several years. Some people choose to self-medicate with hormones to avoid delayed treatment [7], which is also seen in many other countries [8–11].

To ensure equitable care, it should be provided according to need, and hence trans and gender-diverse people should be a prioritized group in healthcare. Instead, they face many barriers when accessing the healthcare system. These disparities in access to care can be linked to cisnormativity, structural discrimination, lack of knowledge, and invisibility [12–16]. Primary care has a major responsibility to improve the health of the whole population, contribute to equity in health and is identified as a key function in improving the health of trans and gender-diverse people [17]. Other than being the obvious first port of call for many health problems, primary care is also often the first point of referral for people seeking gender-affirming care in Sweden. Some trans and gender-diverse people seek gender-affirming care to reduce the gender dysphoria that comes from living in a body that does not match their gender identity. Such care is classified as highly specialized in Sweden and only allowed to be given at gender identity clinics in the country. If support is needed during the waiting time to access such care, that support should, in most cases, be given within primary care. The process of changing legal gender has been made smoother with an updated Gender recognition act from 2025 that no longer requires contact with gender affirming care. The law now requires that the person is living in Sweden, has turned 16 years (with support from the legal guardian until 18 years old) and a letter from a health care provider stating that the persons legal gender does not match their gender identity and that it is assumed the person will continue to live accordingly to this identity in the future. Such letters can be written by doctors, psychologists, psychotherapists and counsellors in any part of health care including primary care. Finally, for trans or gender-diverse people on treatment with hormone injections, primary care is where most people get their injections.

Previous research from other countries on contact with primary care has shown that trans and gender-diverse people have low confidence in primary care providers and that they experience major knowledge gaps and discrimination. It also shows that the focus tends to end up on their trans identity instead of the need for care, and that they may postpone or even entirely avoid seeking healthcare [9, 18–23]. There are also trans and gender-diverse people with positive experiences of primary care, and such experiences seem to increase their confidence and motivation to seek care in the future, as well as being associated with better health [24, 25]. A US survey (*N* = 27 715) among trans and gender-diverse people found associations between better health and having seen a doctor in the previous year, having a primary care physician, and having a primary care physician with trans knowledge [26]. A Canadian survey (*N* = 923) also showed a correlation between feeling comfortable with one’s primary care physician and better health [27]. Thus, trans-competent primary care has the potential to contribute to improved health among trans and gender-diverse people. In a US study, trans and gender-diverse focus group participants described three aspects that defined trans-competent care: respect for trans identities, being treated as a whole person and not just based on one’s trans identity, and partnership with one’s healthcare provider [28].

Studies on trans and gender-diverse people’s experiences of seeking care outside of gender-affirming care in Sweden show results in line with international research, including a lack of knowledge among care providers and the risk of postponing or avoiding seeking healthcare [29–32]. These studies briefly touch upon primary care, but to the best of our knowledge, there is no research focusing on experiences from primary care. This study therefore aimed to explore trans and gender-diverse people’s experiences of primary care in Sweden. The findings can be used to improve care encounters and, by extension, equity in access to primary care.

## Method

As our intention was to explore experiences, we chose to conduct a qualitative interview study. Both authors have extensive experience of working with trans issues, IL primarily from more than 10 years of research and AR from 20 years of lived experience as a trans person and working within non-profit organizations. Both are trained medical doctors but only AR works clinically.

### Study context: Swedish primary care and legal aspects

Healthcare in Sweden is governed through the Health and Medical Service act (2017:30), stipulating that care should be given on equal terms and the one with most need should be given precedence. Primary care in Sweden is funded by the state via taxation, and consists of health centres (vård- eller hälsocentraler in Swedish) run by both public and private healthcare providers. The care is characterized by large units employing a range of different professions. In addition to physicians and nurses, other professions such as specialist nurses, psychologists, counsellors, physiotherapists, midwives, and occupational therapists are more commonly found at primary healthcare centres in Sweden compared to other countries [33]. By law, residents have the right to seek care at any primary healthcare centre they choose, and cannot be denied care. There is also protection against discrimination based on gender identity and expression in Swedish law.

### Recruitment

Advertisements for participation were first disseminated through primary healthcare centres and gender identity clinics in the country, and later through trans organizations (Transammans and Full Personality Expression Sweden) and social media. The final group of participants became aware of the study through Transammans and social media. In Transammans dissemination of the advert, it was stated on their initiative that we had already conducted several studies on trans people’s health and experiences of healthcare and that we had long shown our commitment to trans issues. Inclusion criteria were: (a) previously or currently identifying as a trans or gender-diverse person, (b) being older than 15 years, and (c) having had contact with primary care in the past 3 years. The option of being interviewed via an interpreter from another language was available but was not used in any of the interviews. All participants had to be able to give consent and to complete an interview, but no other exclusion criteria were set. Participants were not remunerated.

### Data collection

Semi-structured interviews were conducted using open questions from an interview guide that was supplemented with follow-up questions based on what the participants shared (see the Appendix). Short summaries were used to make sure experiences were accurately understood. The interview guide was based on previous studies and developed through discussion between the authors. After the initial interviews, the interview guide was revisited without any major changes. Participants were given the choice to be interviewed face-to-face or digitally; four interviews were conducted face-to-face and eight via the Zoom software application. Interviews were conducted in March and April 2024. Interviews were conducted in Swedish.

All interviews were recorded after demographic data (gender identity, age, place of residence, occupation, etc.) were collected in writing. Interviews lasted between 15 and 75 min. Brief notes were taken after the interviews. All participants were offered the opportunity to get in touch or to be contacted after a few days to address any difficult feelings after the interview, and a few of them took advantage of this, resulting in short supporting talks about difficulties in contact with health care. The interviews were transcribed verbatim, two by the authors and the others via a professional service. Those transcribed by others were listened to in full, and the transcripts were corrected.

### Participants

A total of 19 people contacted the research team. One was younger than 15 years and hence was excluded. The remaining 18 were offered an interview; six of these declined without any specific reason given or did not reply, and so interviews were held with the remaining 12. The participants were aged 22–68, lived in both large cities and small rural areas across the country, and represented a variety of identities (four non-binary people, five women (one currently identifying as a “trans woman”), three men (two currently identifying as “trans men”)). One was born in Syria, one in Denmark, and the rest in Sweden. Five had children. Four were receiving a pension or social security benefits and the rest were working or studying. All but one had received, or wished to receive, some kind of gender-affirming care; some had their first contact many years ago and some were currently starting up their care. Reasons for contact with primary care included symptoms such as fever or abdominal pain, mental health issues, check-ups for chronic diseases such as asthma or hypertension, referrals for gender-affirming care, testosterone shots, and administrative reasons such as sick leave. To reduce the risk of traceability, we do not provide a list of participants and their demographics. Quotes will be presented with pseudonyms and the persons gender identity stated.

### Analysis

The interviews were analysed using reflexive thematic analysis according to the six phases proposed by Braun & Clarke [34]. The interviews were read through in its entirety before coding began (phase 1). Initial coding was performed with paper and pencil, after which the codes were entered into Word and then discussed between the authors (phase 2). Early analytical ideas were noted in parallel. In the next step, the codes were transferred to Excel and sorted into preliminary themes (phase 3). These preliminary themes were then printed on paper. Once all codes were sorted, each theme was reviewed individually (phase 4) and discussed by the authors based on coherence within the theme and validity in relation to the whole data set. Preliminary themes were merged and split, and those falling outside the aim of the study were discarded. All transcripts were re-read to see if the themes reflected the data and to ensure that none of the data had been overlooked in the codes. Once themes were defined and deemed to reflect the data well, analyses of all the themes were written, and categorized into five themes (phase 5). Finally, the article was written and the findings put in relation to the conceptual ideas and previous research (phase 6).

## Results

Five themes were identified in the analysis: “Preparations and low expectations”, “Attempts at trans-competent care”, “When trans experiences are not present – from smooth to excluded”, “When being trans becomes a problem – from friction to overt violations”, and “Effects of healthcare encounters – from discomfort to shifted responsibility”. These themes are presented chronologically based on what happens before contact with primary care, what happens during the care encounter, and the effects that occur during and after the encounter. As we will show, this chronology is in reality more of a circular movement, as experiences from previous encounters influence both expectations and the choice of strategies for navigating the encounter. The five themes and examples of codes within each are presented in Table 1.

**Table 1:** Themes and examples of codes

Themes	Examples of codes
Preparations and low expectations	Having low expectations Worrying before visits Knowing it could be worse Preparations before care Finding care Avoiding care
Attempts at trans-competent care	Attempts at trans-competence One’s own positive reactions
When trans experiences are not present – from smooth to excluded	Smooth meetings Trans experiences are not noticed Invisibility of identity Misgendering Excluding experiences Limiting medical records Easier after transitioning Preparedness to openness
When being trans becomes a problem – from friction to overt violations	Lack of competence Uncomfortable caregivers Being trans gets too much attention Violations Being denied care
Effects of healthcare encounters – from discomfort to shifted responsibility	Uncomfortable care seekers One’s own negative reactions Protesting Choosing not to protest Choosing not to be open External support

### Preparations and low expectations

This first theme illustrates how the participants’ low expectations and anxieties about primary care encounters led to preparations and efforts to find trans-competent care or to postpone or avoid seeking care altogether.

Based on the level of competence in society along with their own and others’ previous care experiences, the participants had low expectations of knowledge among primary care providers about trans and gender-diverse people. They reasoned that keeping their expectations low would reduce their disappointment if shortcomings arose. One participant described how their experiences from society followed them to the healthcare encounter:

*It’s all about having tried [to be recognized as a non-binary person] a number of times*,* and it doesn’t even have to be in healthcare. It’s about having an overall understanding of the public*,* how far the public has come in terms of pronouns*,* for example. (Dex*,* non-binary)*

The participants prepared mentally for the physical examinations that might be required, contacted the clinic and stated their pronouns in advance, and chosed clothes they believed would increase the chances of a good encounter. A few of them expressed confidence in the healthcare system in general, but anxiety about seeking healthcare was consistently expressed. Their nervousness typically concerned which reactions they might encounter, as Karin said:

*And there’s always the fact that you don’t know what kind of treatment you’ll get. You don’t know what this person will think or feel about your body. (Karin*,* woman)*

The participants made efforts to find clinics and providers with the best trans-competence. For example, they sought out specialized or LGBTQ-certified clinics, or asked to see the healthcare provider in the unit with the most knowledge about trans issues. One person had changed her primary healthcare clinic after being refused care at her first one when her trans identity became known. Several participants postponed or completely avoided seeking care out of fear of poor treatment. The following quotation illustrates how the decision to seek care was preceded by doubt and hesitation:

*I often think about it several times*,* but when it comes to the point where I feel I have to*,* then I do it. But it’s not so easy*,* I think about it over and over again before I take that step [of seeking care]. (Yasmin*,* trans woman)*

Gynaecological problems were reported as particularly difficult for trans men and non-binary participants assigned female at birth to seek care for, partly because of the extra vulnerability of the examination. Another difficulty in this context was that the waiting area could be a stressful place to be in as a trans man or gender-diverse person, due to its potential to reveal one’s reason to be there and hence to reveal one’s trans status (for example, a waiting area for midwife appointments).

### Attempts at trans-competent care

Most participants had both positive and negative experiences of contact with primary care, and so some attempts at trans-competent care were visible in the interviews, which is the focus of this theme. We specifically asked about positive experiences, and examples emerged of care where people were asked about their pronouns, and where they felt respected and treated with care and attention. The professionals involved in these examples were physicians, nurses, assistant nurses, physiotherapists and midwives. These encounters were described as exceptions, but they created trust, and made the participants feel satisfied and recognized. The efforts made by these care providers were appreciated; for example, one participant described how it felt when a care provider showed them the medical record from their previous meeting and was keen to make sure that the correct pronoun was used:

*Then they had it printed out*,* “This is what I’ve written. Is it okay?” That was a bit anxious*,* but still really trying. And they’d written pronouns in maybe eight different places and it was right in six places. And we [the participant and their partner] just went*,* “There too.” “Okay*,* I’ll go and change it.” And fixed it. “Maybe we should look at what I wrote from today before you leave.” It was pretty cute*,* after all. (Dex*,* non-binary)*

The quotation above shows a willingness to do the right thing on the part of the care provider, but also their inability to write the correct pronoun in the record on their own. The participant noticed this anxiety, and time had to be taken from the visit to read through the record together. This also meant that the responsibility for ensuring correctness in the medical record was placed on the patient, which is usually not the case. Despite this, the experience was described in positive terms, reflecting both the expectations of the care and the other experiences with which it was compared. This is further illustrated in the following two themes.

### When trans experiences are not present – from smooth to excluded

This theme describes how cisnormative encounters, i.e. when the trans experiences were not noticed, were sometimes equivalent to uneventful encounters, but other times led to trans and gender-diverse people’s existence being made invisible in primary care. Examples of the latter included instances in which care seekers were misgendered, and when care providers were not receptive to information about the participants’ trans identity or trans history.

Many of the primary care encounters with different professionals described by the participants were characterized by the care provider assuming that the patient was cisgender, and their trans or gender-diverse identity was never discussed. Some of these encounters were described as smooth, and the participants felt satisfied when the care provider’s assumption of their gender identity was correct and/or when their trans experience was not relevant, as Yasmin said:

*It’s probably*,* I don’t know if that’s the case*,* but that’s how I feel; it turns out well when I don’t*,* when the situation doesn’t require me to tell my whole story. (Yasmin*,* trans woman)*

Several participants described a willingness to come out if they thought it was relevant or when care providers asked or showed interest. However, examples were given of trying to share information about their trans experience when they considered it relevant, by talking about their hormone treatment or surgeries, without the care providers seeming to register the information. This may indicate that the care providers’ presumption of the patient being cis was so strong that it was not disturbed even when the patient said otherwise. The participants also highlighted that narrowly worded questions from care providers might not get full answers, so information might be missed. For example, follow-up surveys with only two gender categories to choose from ran the risk of missing non-binary respondents due to not allowing them to indicate their gender.

When healthcare providers’ cisnormative assumptions about gender identity were not correct, the participants were misgendered. Misgendering happened most often in the text of medical records, but it also occurred on the phone and face-to-face. The participants particularly described experiences of being misgendered in face-to-face encounters when seeking care with their children, when they were addressed in the third person and referred to with gendered parental words such as “mum” or “dad” when the care provider was talking to the child. These participants also pointed out that it felt more important to try to correct the care provider when their child was present, as a way of standing up for themselves in front of their child.

Several participants, especially non-binary people, spontaneously raised feelings of invisibility and exclusion as a general response to the first open question in the interview about what it was like to encounter primary care as a trans or gender-diverse person; for example: “My feeling about meeting primary care is that there is no place there for me to be a trans person” (Dex, non-binary). Misgendering and the feeling of not being seen as oneself can be understood as an exclusion of trans experiences from primary care.

The participants expressed both disappointment that the medical record only had two gender categories, and the hope that in the future there would be the possibility of it including information about gender identity and pronouns. Several participants said that it was easier to seek care after receiving gender-affirming care because their self-esteem was higher; as one participant said: “Because now I am somehow comfortable with my body anyway” (Oskar, man). The same applied after changing name and social security number, as the likelihood of being seen as oneself was then greater.

### When being trans becomes a problem – from friction to overt violations

This theme shows the participants’ experiences of when being trans becomes a problem; for example, when transphobia and violations were noticed, or care providers became uncomfortable. Moreover, sometimes knowledge gaps were revealed that led to patients being forced to explain matters in order to receive care, as well as situations where trans or gender-diverse experiences were given too much focus by curious care providers, and when patients were denied care because of their identity.

When being trans became present in the healthcare encounter, transphobia could also manifest itself. Healthcare providers from different professions were perceived as uncomfortable, as avoiding the topic, or as physically distancing themselves from the patient. One participant described their first visit to a clinic:

*Let’s take my first contact with a doctor. As she gradually understands who I am*,* she pulls further and further away until she sits in the corner looking like a terrified mouse. And that was not a very uplifting experience. (Anna*,* woman)*

Significant gaps in care providers’ knowledge were revealed, and the participants were forced to explain to and educate their care providers in order to receive care. These knowledge gaps ranged from what it means to be trans and how gender-affirming care works, to handling a new social security number or protected personal data. These knowledge gaps were reported from different professionals, such as physicians, nurses, and reception staff. Trans or gender-diverse experiences were sometimes given too much focus, and care providers were perceived as being too curious and as asking awkward questions.

*But it’s like*,* there will be an extra round of questions*,* ten minutes more about something that isn’t part of the agenda*,* or not why I’m there at that point. And then you see the confusion in their eyes when they’re suddenly trying to figure out who you really are. (Karin*,* woman)*

Overt violations in the form of ridicule, disrespect, and questioning were also present in the participants’ stories, such as when a participant booked an appointment on the phone and then received a call:

*Five minutes later she calls back and she’s just*,* “But the social security number you gave belongs to a man*,* who are you booking an appointment for?” (Yasmin*,* trans woman)*.

Several participants had unsuccessfully tried to get help with blood tests while self-medicating with sex hormones, being met only with no interest or dismissive comments from the physicians, such as claiming a referral was needed or competence was lacking. Some had also been completely denied care when trans issues came up. For example, one participant called her clinic to make an appointment and wanted to inform them that she would soon be getting a new social security number due to her change of legal gender:

*I explained a bit like*,* that was before I got my new social security number*,* so I explained a bit “So you know I will have a new social security number and…” then everything shifted*,* they treated me well at first but then everything just shifted*,* and it was just no*,* “We don’t have time to see you*,* you have to contact another clinic.” (Miranda*,* woman)*.

The participant above had made an effort to inform the clinic about her upcoming change of social security number to facilitate her future care, but was then instead completely rejected by the clinic.

### Effects of healthcare encounters – from discomfort to shifted responsibility

This theme captures the effects when the participants encountered cisnormativity and transphobia in primary care, which included both negative emotional reactions and resistance strategies. These resistance strategies included protesting, actively choosing not to speak out, not being open in order to avoid friction and conserve energy, and seeking support from the trans community and family members when care was not supportive or when knowledge was lacking.

Both cisnormative assumptions and transphobic behaviour created discomfort and a range of negative emotional reactions in the participants. They felt uncomfortable when they realized that information was missed, disappointed and surprised by lack of knowledge (“surprised that I had to explain it yet again” [Peter, trans man]), surprised by poor treatment (“it’s more of a surprise when I think it’s going to be fine and then something happens” [Eli, non-binary]), tired of having to explain themselves, and angry about being made to feel invisible. Furthermore, they reported feelings of dissatisfaction, hopelessness, and powerlessness. One participant reflected on the feeling of invisibility and how it felt to never be asked what their gender identity was:

*I mean it’s tiring. It’s very tiring. If I think about it too much*,* I get angry. Because you believe care providers should know better. But they don’t. They don’t know anything. (Jo*,* non-binary)*

Many of them had tried to speak up and correct a care provider, often without success, and many therefore actively chose not to speak up as a way of saving their own energy and avoiding the risk of having to take time away from the encounter to explain themselves. Another common strategy was to actively choose not to be open about their trans or gender-diverse identity, to avoid the risks that come with openness. They braced themselves and took on a role, expecting not to be seen as themselves in the healthcare encounter.

*That’s why I just dive in*,* say my name*,* don’t give a fuck about pronouns*,* do whatever needs to be done*,* never see that doctor again*,* and know the medical record will be wrong. (Dex*,* non-binary)*

The participants might also withhold medical information such as being on hormone treatment to avoid having to be open, despite being aware of the risks involved. Another coping strategy was to get support in the form of medical knowledge from relatives and the trans community; for example, one self-medicating participant said: “Even if you don’t know much, there are very big online communities.” (Felicia, woman). This illustrates how the responsibility for ensuring appropriate medical knowledge and care was shifted from care providers to care seekers.

## Discussion

Trans and gender-diverse people in Sweden have varying experiences of healthcare encounters when seeking primary care. A few participants in this study had a generally high level of trust and satisfaction with the healthcare system, and the interviews revealed some individual examples of attempts at trans-competent care in which participants were allowed to define their gender themselves, were given time and space, and were treated with respect. However, as has been seen in previous studies, many participants shared experiences of lack of trust and reported negative reactions among care providers [18].

The presence of cisnormativity in primary care was clear in this study. The participants’ experiences of being misgendered, made invisible and being excluded, and care providers not being receptive to all information indicate that care providers of different professions seem to unconsciously assume that they have a cis person in front of them as a patient. This has also been seen in previous studies [9, 21, 28, 30]. In addition to the patient not feeling seen, this situation entails a risk of errors in diagnosis and treatment, as medical information is lost. The healthcare provider thus risks causing distress and harming the patient in several ways without being aware that this is happening. Some cisnormative expressions described by our participants were connected to built-in institutional issues, such as when medical records could not accommodate the correct pronouns, or lack of education in undergraduate programmes, while others were more connected to individual care providers. This illustrates the importance of actions on different levels, both systemic and individual, to challenge cisnormativity in primary care and to contribute to more equitable access to appropriate care.

When being trans becomes present in the care encounter, it often creates friction. As in previous research [18, 21, 29–31, 35], knowledge gaps meant that our participants were forced to educate their care providers in order to receive care. Healthcare providers of different professions were perceived as uncomfortable and distant, insults occurred, and patients were sometimes denied care. These discriminatory acts, also reported in previous research [18], range from cisnormativity to transphobia. When a healthcare provider backs into a corner or hangs up the phone when it becomes apparent that the patient is trans, this is an example of transphobia; that is, a more conscious individual process based on fear or hatred [1].

A Canadian research group [35] concluded that both cisnormative behaviours, where health professionals are unaware of what they are doing, and overtly transphobic actions risk devastating consequences when the patient’s care needs are not met, and harm is inflicted. This is supported by our study. In line with other studies [20, 22, 23], we can see that trust in primary care among trans and gender-diverse people is low, which can be understood as a consequence of cisnormativity and transphobia encountered earlier in life, both in healthcare and in society at large. Due to a low level of trust in healthcare, participants in this study put a lot of energy into creating good opportunities for good care encounters, including mental preparation, considering whether it was worth seeking care at all, seeking out specific clinics or care providers, and presenting themselves in advance. This is work that no patient should have to do and illustrate how access to care is not equitable. A quantitative report with 800 trans and gender-diverse respondents in Sweden from 2015 showed that trans and gender-diverse people reported much less trust in healthcare compared to the overall population (43% versus 69%) [2]. The present findings also illustrate a shift in the responsibility for creating a good encounter from care providers to the patient. Similar strategies of navigating care by seeking out particularly trans-competent clinics and providers, as well as avoiding seeking care, have been reported previously [20, 21, 36].

Once in the healthcare encounter, trans and gender-diverse patients have little power to influence what is happening. Several of our participants had tried to speak up and make corrections when healthcare providers used the wrong pronouns, for example, but often the healthcare providers did not even realize that they were being corrected. This can be interpreted as cisnormativity being so strong that it was not shaken by new information, probably also reinforced by the power imbalance between patient and care provider. Many participants therefore chose strategies such as not speaking out and assuming a role where their identity was not recognized (for example, letting care providers assume they had another gender identity), in order to seek care and minimize the risks of negative consequences. This strategy is sparsely described in other research, with one exception being a previous Swedish study [31]. The reason for this might be that other studies have included a lower proportion of non-binary participants, and in our study, it was mainly these participants who used the strategy of not being open with their identity. Not being able to be yourself as a patient carries an obvious risk of poorer quality of care, both with regard to the effect on the patient’s trust in the care provider and their compliance with advice or treatment, and because information might be lost and medical decisions made on insufficient grounds.

The participants expressed the desire to be treated respectfully as whole persons, alongside increased trans-competence and education; other studies have reported similar findings [19, 28]. Wishes for help with blood tests for monitoring self-medication or for the physician to take over the prescription of hormones also emerged in our study. According to the guidance from the National Board of Health and Welfare for care of gender dysphoria, healthcare providers should “consider the danger of self-medication without monitoring and what the potential risks may be if a healthcare professional chooses not to take over the hormone treatment” [7, p. 48]. Despite these guidelines, our interviews included examples of patients who found it difficult to gain acceptance even for blood tests.

Arguably, it is the responsibility of the care provider to evaluate whether a patient is being harmed by any medical treatment that they become aware of, and it would be desirable if more physicians could take over such prescribing to ensure adequate dosing and reduce the risk of side effects. The desire to have hormones prescribed by one’s general practitioner is also reported in studies from other countries [20, 21, 37]. Moreover, gender-affirming care is increasingly being provided in primary care in several countries, which is argued to provide a shorter path to treatment as well as preserving the patient’s self-determination [18]. In Sweden, developments are moving in a different direction, as centralization has increased since the National Board of Health and Welfare decided in 2020 that care for gender dysphoria should be classified as national highly specialized care [38].

Since our results are similar to the findings of previous studies, we consider it reasonable to say that the present results are transferable to primary care in countries with similar healthcare settings. It is also likely that cisnormativity and transphobia as shown in this study have an impact on healthcare in general, and so the results can be used to improve general healthcare and equity in health for trans and gender-diverse people. Such improvement would require increased trans-competence for all professionals in primary care, which can be achieved through curricular changes in undergraduate programmes and continuing education for staff. Structural changes are also needed; for example, making it possible to record the patient’s correct (preferred) name and pronouns in their medical records.

To ensure that interventions have the best possible effects, involving trans organizations in development work is of the utmost importance [39]. Trans and gender-diverse people are a heterogeneous group with different needs, and so an intersectional understanding is necessary to reduce inequity [26]. However, if the focus of development work is on improving care for the most marginalized who face multiple oppressions, care will be more equitable and better for all.

### Strengths and weaknesses

The participants in this study represented a wide range of trans and gender-diverse people in Sweden, with different ages, identities, and places of residence across the country. Both authors have a long-term involvement in trans issues from research, lived experience and non-profit organizations, which we believe has been a strength of the study. Our personal contacts in trans organizations made it easy for us to distribute advertisements for recruitment. Most participants registered their interest via the trans organization that told them about our commitment and knowledge of the issues, and this might have made a difference to their interest in taking part. Our hope is that it increased the confidence of our interviewees, as they knew we would have an understanding of their experiences. There may, however, have been people who chose not to participate in the study because of previous or current contact with us.

To ensure that data reflects participants own experiences open questions with summaries during interviews to check that experiences were correctly understood was used. The data, codes and themes were analysed by both authors and interpretations compared to further enhance credibility. To reduce the risk that knowledge of previous studies and hearsay about negative experiences of healthcare contact would colour the analysis, positive experiences were actively sought in the interviews and looked for in the coding.

The fact that all participants were recruited via a trans organization and social media means that we did not reach any potential participants who had no contact with other trans and gender-diverse people. The experiences of contact with primary care should not differ to any great extent, but the opportunities for support from the community are clearly poorer, and so the effects of a lack of competence might also be worse. We were not able to analyse effects of racism due to few participants of minoritized ethnicities.

### Future research

As a large proportion of the existing studies on trans and gender-diverse people’s experiences of primary care are qualitative, more quantitative data of for example care seeking patterns, avoidance, compliance and health outcomes would be valuable. Research aimed at evaluating training programs or other ways of improving trans-competence in, and improved access to, healthcare would be of great value. To help shape education and training, studies could examine which supportive actions from primary care providers can improve trust in healthcare, decrease postponement and avoidance, and hence improve health among trans and gender-diverse people. It is essential that trans organizations are involved in the work of formulating research questions and developing competence-enhancing measures, in order to achieve the best and most relevant results possible.

## Conclusion

Findings show that participants’ experiences with primary care in Sweden were influenced by cisnormativity, leading to invisibility, exclusion, misgendering, and a lack of knowledge. Experiences of discomfort on the part of care providers, ridicule, disrespect, and denial of care imply the presence of transphobia. These barriers result in trans and gender-diverse people experiencing low levels of trust, having to navigate their care without support, and not having their healthcare needs met. There is an urgent need for increased trans competence and allyship with trans communities to ensure equitable access to healthcare. Training and further research should be developed in close consultation with trans organisations to address these disparities.

## Supplementary Information

Supplementary Material 1.

## Data Availability

The interview data that support the findings of this study are not openly available due to reasons of ethics and sensitivity but might be available from the corresponding author upon reasonable request and approval from ethical review authorities. Data are stored in encrypted services at Umeå University.
