# Supplementary material for: Trans and gender-diverse people’s experiences of primary care in Sweden – a qualitative study
Source: BMC Prim Care. 2025 Nov 15;26:364. doi: 10.1186/s12875-025-03100-9 (PMC12619444; doi:10.1186/s12875-025-03100-9)
Supplement: Supplementary file 1 — Supplementary Material 1. [file 12875_2025_3100_MOESM1_ESM.docx]

# Interview guide (Translated from Swedish)

The interviews will focus on transgender people’s experiences of primary health care. The guide includes exploratory questions intended to stimulate conversation and encourage the participant to reflect on and share their experiences. Follow-up questions will be asked based on the participant’s answers. Throughout the interview, the participant’s boundaries will be respected — for example, by being attentive to topics they may not wish to talk about.

Demographic information (not recorded, only noted):

Gender identity? Pronoun? Sexual orientation? Age? Occupation? Place of birth? Where do you currently live? Do you live with anyone? Partner(s)? Do you have children? Any gender-affirming care?

(Start recording) Opening question:

How would you describe what it’s like to encounter primary health care as a transgender person?

1. What does good health mean to you? How would you describe your own health in relation to that definition? In what ways do you think being transgender affects your health today? How has that changed over time? Have you sought care because of your health situation?
2. Where do you usually turn when you have health concerns? Why do you go there?
3. What experiences do you have with primary health care? What made you seek care in primary health care? How do you experience the support and care you received? How have you been treated? Do you feel there was any competence related to trans issues? Do you prepare in any particular way before visiting primary care?
4. Can you describe a healthcare encounter in primary care? How did you make contact? Who did you meet? What worked well? What worked less well?
5. Do you have an example of a healthcare encounter that went really, really well? What do you think made it such a positive experience? Do you have other experiences that felt especially empowering or affirming in a healthcare setting?
6. Are there healthcare encounters you postpone or avoid? Why?
7. Have you been in contact with primary care to get a referral for gender-affirming care? How did that go?
8. What are your main needs and wishes in relation to health care? How do you think primary care could be improved?

Closing questions:

1. Is there anything you would like to add or bring up that we haven’t touched upon?
2. (Stop recording) How did you experience the interview?
